# Increased Hypothalamic Projections to the Lateral Hypothalamus and Responses to Leptin in Rat Neonates From High Fat Fed Mothers

**DOI:** 10.3389/fnins.2019.01454

**Published:** 2020-01-31

**Authors:** Lyla Kelley, Silvanna Verlezza, Hong Long, Mary Loka, Claire-Dominique Walker

**Affiliations:** ^1^Douglas Mental Health University Institute, Montreal, QC, Canada; ^2^Department of Anatomy and Cell Biology, McGill University, Montreal, QC, Canada; ^3^Integrated Program in Neuroscience, McGill University, Montreal, QC, Canada; ^4^Department of Psychiatry, McGill University, Montreal, QC, Canada

**Keywords:** maternal programming, high fat diet, neonatal, leptin, lateral hypothalamus, orexin

## Abstract

The lateral hypothalamus (LHA) is a central hub in the regulation of food intake and metabolism, as it integrates homeostatic and hedonic circuits. During early development, maturing input to and output from the LHA might be particularly sensitive to environmental dietary changes. We examined the effects of a maternal high fat diet (HFD, 60% Kcal in fat) on the density of hypothalamic projections to the orexin (ORX-A) field of the LHA in 10 day-old (PND10) rat pups using retrograde labeling with fluorescent microspheres. We also compared responsiveness of phenotypically identified LHA neurons to leptin administration (3 mg/kg, bw) between pups from control (CD) or high fat (HFD) fed mothers on PND10 and 15-16, at the onset of independent feeding. HFD pups exhibited a higher density of LHA projections (*p* = 0.05) from the ventromedial hypothalamus (VMH) compared to CD pups and these originated from both SF-1 and BDNF-positive neurons in the VMH. Increased circulating leptin levels in HFD pups, particularly on PND15-16 was consistent with enhanced pSTAT3 responses to leptin in the orexin (ORX-A) field of the LHA, with some of the activated neurons expressing a GABA, but not CART phenotype. ORX-A neurons colocalizing with pERK were significantly higher in PND15-16 HFD pups compared to CD pups, and leptin-induced increase in pERK signaling was only observed in CD pups. There was no significant effect of leptin on pERK in HFD pups. These results suggest that perinatal maternal high fat feeding increases hypothalamic projections to the ORX-A field of the LHA, increases basal activation of ORX-A neurons and direct responsiveness of LHA neurons to leptin. Since these various LHA neuronal populations project quite heavily to Dopamine (DA) neurons in the ventral tegmental area, they might participate in the early dietary programming of mesocorticolimbic reward circuits and food intake.

## Introduction

The lateral hypothalamus (LHA) is central to the regulation of food intake as it integrates incoming metabolic signals from several hypothalamic nuclei with the hedonic regulation of food intake, involving the mesolimbic circuit and in particular, dopamine (DA) activity in the ventral tegmental area (VTA). Homeostatic hypothalamic nuclei such as the arcuate nucleus (ARC), ventromedial hypothalamus (VMH), dorsomedial hypothalamus (DMH), and hypothalamic paraventricular nucleus (PVN) project either directly or indirectly to the LHA (Hahn and Swanson, 2015) and together with multiple cortical, subcortical and brain stem inputs, regulate LHA activity (Bonnavion et al., 2016). In the LHA, multiple cell types are known to project to the VTA where they control dopamine and GABA interneuron firing activity and among these, several harbor functional leptin receptors (LeptR_*b*_), including Neurotensin (Nts), Cocaine and Amphetamine Regulated Transcript (CART) neurons as well as different populations of GABA-containing neurons projecting to VTA GABA neurons (Nieh et al., 2016; Stuber and Wise, 2016; Godfrey and Borgland, 2019). Orexin-A (ORX-A) containing neurons in the LHA directly project to VTA dopamine neurons and stimulation of these cells has been shown to increase VTA DA firing activity and increase food intake (Tyree and de Lecea, 2017). While ORX-A neurons projecting to the VTA do not contain leptin receptors (Leinninger et al., 2009; Leinninger, 2011) they are indirectly inhibited by leptin in an energy-dependent state, whereby high fat feeding dampens the inhibitory effect of leptin on these neurons in adult mice (Liu et al., 2017), resulting in increased food intake.

In rodents, connections between “homeostatic” hypothalamic nuclei and the LHA are not fully established at birth and mature over the first weeks postnatally, making them susceptible to changes in the perinatal nutritional environment (Muscatelli and Bouret, 2018). Projections from the ARC to the DMH mature by postnatal day (PND) 6, whereas those reaching the PVN and the LHA are observed between PND8-10 and PND12, respectively (Bouret et al., 2004a). In neonatal mice, projections from the DMH to the PVN and LHA are fully established by PND6, followed by projections from the VMH to the LHA, which develop by PND10, prior to those from the ARC (Bouret et al., 2004b). While the development of hypothalamic homeostatic connections has been well studied, much less is known on the development of efferent projections from the LHA to the VTA. Using retrograde tracing, we previously showed that functional connections already exist between these two nuclei by PND6 and that some of these originated from ORX-A neurons (Gjerde et al., 2016). Thus, by PND10, the LHA is already connected to both homeostatic and hedonic centers even though independent food intake does not start until PND17 in rat pups from control mothers or PND16 in pups from high fat diet (HFD)-fed mothers (Doerflinger and Swithers, 2004). Similarly to the earlier onset of independent feeding, central food intake mechanisms, food seeking behavior, and food preferences are altered by changes in the perinatal nutritional environment, increasing vulnerability to adult metabolic diseases (Bouret, 2012; Ribaroff et al., 2017; Romaní-Pérez et al., 2017). The metabolic hormone leptin inhibits food intake and increases energy expenditure in adult rodents. Yet, during perinatal life, it may be critical to developmental programming because of its important role in neurite outgrowth and synapse formation (Bouret et al., 2004b; Bouret, 2010). Circulating leptin concentrations in the offspring are also significantly increased when mothers are fed a HFD (Trottier et al., 1998).

During hypothalamic circuit maturation, leptin’s role on the development of neuronal projections might supersede its primary role to suppress food intake. The production of leptin-induced intracellular signaling molecules such as phospho-STAT3 (pSTAT3) and phospho-ERK (pERK) (Kwon et al., 2016) might not only indicate the presence of functional LeptR_*b*_ receptors, but also provide an index of tissue responsiveness and/or resistance to increased circulating leptin levels. We previously documented a gradual and site-specific emergence of tissue responsiveness to leptin in naïve neonates (Naef et al., 2014; Gjerde et al., 2016) with significant pSTAT3 production in the ARC already by PND10 and by PND16 in the VTA. In the LHA, increases in pERK were documented on PND16. However, it is currently unknown whether exposure to a HFD in early neonatal life modifies LHA responsiveness to leptin and induces early resistance to leptin as observed in adult rats (Matheny et al., 2011). Given the ability of maternal HFD feeding to increase leptin levels in offspring during the maturation of food intake circuits and the central role of the LHA in coordinating homeostatic and hedonic regulation of food ingestion, the aim of our studies were to determine whether perinatal maternal HFD feeding influences the density of hypothalamic projections to the LHA and modifies responsiveness to leptin in phenotypically identified LHA neurons. Our results show that VMH projections to the LHA are significantly increased in PND10 neonates nursed by HFD mothers and that contrary to the adult condition, leptin still elicits a robust intracellular response in LHA neurons from HFD preweaning offspring at the time of the onset of independent feeding.

## Materials and Methods

### Animals

Pregnant Sprague–Dawley females (Charles River Laboratories, Inc., St. Constant, QC, Canada) were obtained on gestation day (GD) 13–14 and housed under controlled conditions of light (12 h light-dark cycle), temperature, and 70–80% relative humidity. All rats were provided food and water *ad libitum*. On the day of arrival, half of the mothers were placed either on a chow diet (CD; Teklad Global diet, 6% kilocalories (Kcal) from fat; Harlan Laboratories) or a HFD [Teklad diet (TD) 06414, 60% Kcal from fat; Harlan Laboratories, Indianapolis, IN, United States] supplemented with peanut butter (approximately 30 g per day; 72% Kcal from fat, 6.7 Kcal/gram) to ensure higher caloric consumption between gestation day (GD)13–14 and postpartum day 15–16 (Table 1). We chose to expose mothers to the HFD during the last week of gestation and throughout lactation in order to (1) target the period of hypothalamic nuclei birth and development (between E13-19 in rats) and circuit maturation in the fetus/neonate and (2) avoid the potential confounding effects of maternal obesity that develops when dams are fed a HFD during the entire pregnancy. The day of parturition was considered PND 0 and on PND1, litters with both male and female pups were culled to either 8 (for retrograde tracing experiments) or 10 pups per mother (for immunohistochemistry experiments). Animals were maintained on their diet until pups were sacrificed at either PND9-10 or PND15-16. Body weight and food intake was recorded on various gestational and postpartum days. All procedures were reviewed and approved by the Animal Care Committee at McGill University and followed ethical guidelines from the Canadian Council on Animal Care (CCAC).

**TABLE 1:** Nutritional information for the control (CD) and High fat (HFD) diets.

**Nutritional information**	**CD: Teklad 18% protein diet**	**HFD: Teklad 60% fat diet**
% Kcal from fat	6.0%	60.3% (37% saturated, 47% monounsaturated, 16% polyunsaturated)
% Kcal from protein	18.9%	18.3%
% Kcal from carbohydrates	75.1%	21.4%
Kcal per gram	3.3	5.1

### Retrograde Tracer Injections in the LHA ORX-A Cell Field of Neonates

**FIGURE 1:**
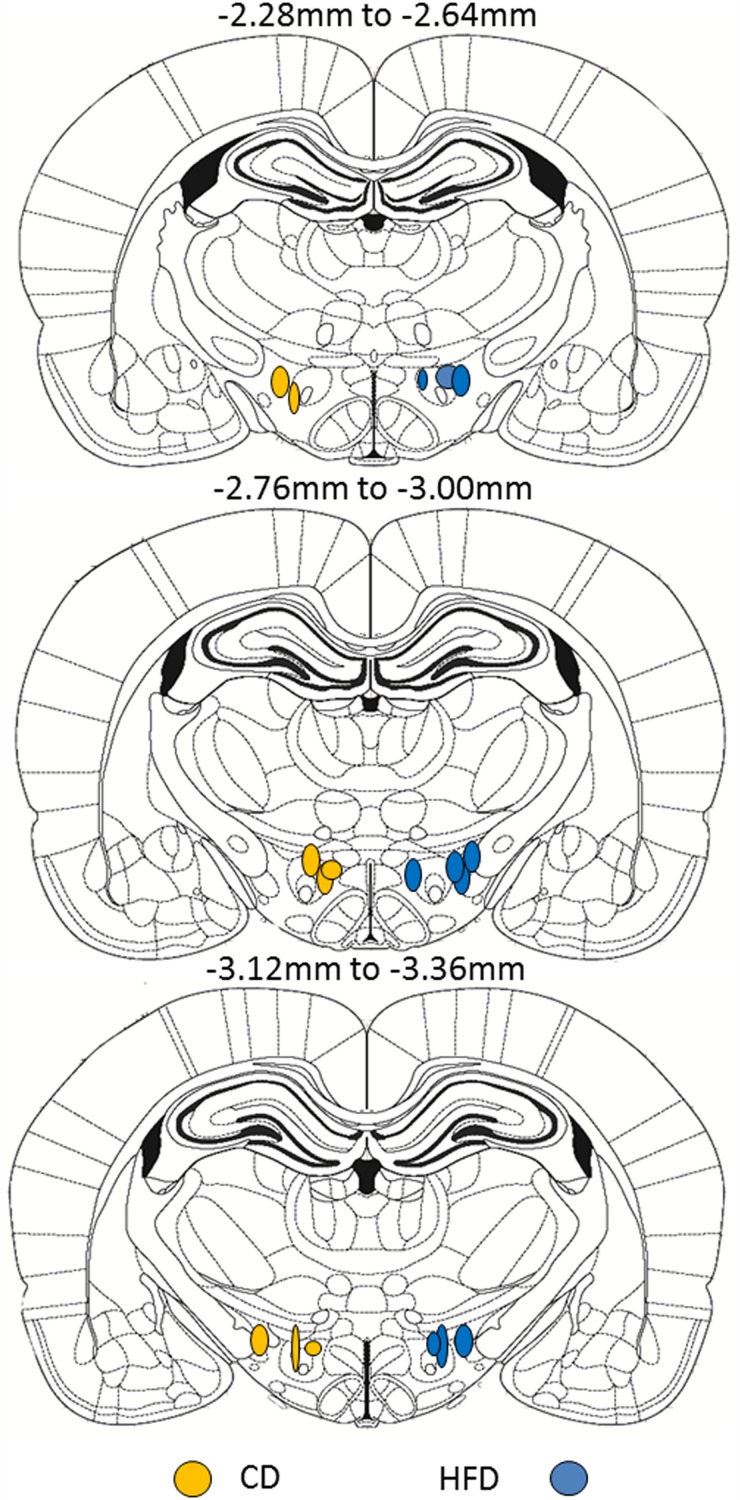
Schematic representation of the site of fluorescent microsphere injections in PND5-6 rat pups from either CD (left side, *n* = 8) or HFD (right side, *n* = 10) litters. Each circle represents one animal and the antero-posterior (A/P) level of injection is indicated according to the atlas of the rat brain from Paxinos and Watson (2005). Within a diet group, injections were randomized between left and right LHA and were only considered accurate within the A/P coordinates shown. For ease of illustration and comparison between diet groups, all injections in CD pups are displayed on the left and injection sites in HFD pups are displayed on the right. Because of the relatively small number of animals per diet group, we did not distinguish between medial and lateral injection sites.

On PND5-6, pups from both diet groups (CD: *n* = 8, average 16.2 g; HFD: *n* = 10, average 17.8 g at time of injection) were anesthetized with isoflurane and placed in a stereotaxic frame with neonatal rat ear bars and anesthesia adaptor (Kopf Instruments, Tujunga, CA, United States). Once anesthetized, the skull of the pup was aligned using bregma and lambda as reference points for the coordinates. Injections were made to target the medial and lateral LHA ORX-A field between the adult coordinates of −2.28 and −3.36 mm anterior/posterior (A/P) from the bregma (Paxinos and Watson, 2005). These adult coordinates were adapted by a factor of 0.663 (and further adjusted based on trial and error in preliminary surgeries) to make injections to neonatal pups (Gjerde et al., 2016). The resulting coordinates used for targeting the medial and lateral portions of the LH ORX cell field were the following: A/P −1.99 to −2.6 mm, lateral: 0.8–1.2 mm, and ventral: −5.95 to −6.08 mm. Red fluorescent fluorospheres (20 nl, #F-8793; Invitrogen, Life technologies Inc., Burlington, ON, United States) were injected using a 0.5-μl Hamilton neurosyringe and a Harvard nanopump mounted on the stereotaxic arm (Harvard Instruments, St-Laurent, QC, Canada). Fluorospheres were injected at a rate of 10 nl/min, and the syringe was left in place for another 5 min before it was slowly removed from the injection site over 1–2 min. The skin was sutured using surgical silk, and pups were returned to their mother only after reaching full consciousness. Four days after microinjection, pups were anesthetized with ketamine/xylazine (subcutaneous injection 0.1 ml/kg body weight) and perfused with ice-cold 0.9% saline (50 ml) followed by 200 ml of 4% paraformaldehyde (PFA) in 0.1 M phosphate buffered saline (PBS), pH 7. Before perfusion, a blood sample was collected on EDTA via cardiac puncture to determine plasma leptin levels. Blood samples were centrifuged (13,000 rpm, 5 min, 4°C), and the plasma was collected and stored at −20°C until leptin assays were performed. Brains were post-fixed for 2 h in 4% PFA-PBS and transferred to 20% sucrose in 0.1 M phosphate buffer (PB) overnight before being frozen on dry ice and stored at −80°C until slicing. Brain tissue was sliced on a cryostat at 50 μm in two series to include at least the LHA (injection placement), VMH, and DMH regions. All slides were coverslipped with DAPI mounting medium (Vector Labs, Burlingame, CA, United States) and kept in the dark to preserve fluorosphere fluorescence until imaging. Each brain was examined for appropriate injection placement to the LHA ORX-A neuronal field, and was only taken for further image analysis if the bottom of the injection fell between the appropriate coordinates as previously indicated (see Figure 1 for injection placement). Images were acquired for both the injection site and the projection neurons.

### Identification of VMH Neurons Projecting to LHA in Neonates by RNAscope

Brain sections adjacent to those used for estimating projection density were used to phenotype the VMH neurons that send LHA projections (colocalizing with fluorospheres). RNAscope *in situ* hybridization (ACDBio, Newark, NJ, United States) for SF-1 and BDNF was performed on tissue sections from the neonatal VMH containing fluorospheres from correct LHA injection sites. Procedures were adapted from the manufacturer instructions. Briefly, VMH sections (50 μm) were transferred to negatively charged slides, incubated for 1 h at 60°C and post-fixed in 4% PFA for 1 h at 4°C. Sections were dehydrated by successive ethanol rinses and incubated for 10 min with 1% H_2_O_2_. After washing (manufacturer wash solution), sections were incubated with RNAscope Protease III for 10 min at 40°C followed by probe hybridization at 40°C for 2 h. After washing, sections were kept overnight at room temperature in 5x SSC buffer. On the next day RNAscope amplification steps were performed prior to HRP developing (15 min) of the signal using specific fluorophores such as Opal520 (BDNF) and Opal 690 (SF-1) exposure for 30 min. Sections were washed and coverslipped with DAPI containing mounting medium (Thermo Fisher Scientific, Canada). Images were acquired with an Olympus FluoView FV-1200 confocal microscope.

### Leptin Treatment and Tissue Collection

On PND10 or 15-16, male and female pups from either the CD or the HFD mothers were randomly assigned to receive an intraperitoneal (0.1 ml) injection of either vehicle (0.9% saline) or leptin (3 mg leptin/kg BW; PeproTech, Rocky Hill, NJ, United States). Pups were returned to their mothers after injection and 1 h later, they were perfused transcardially with 50 ml of ice-cold 0.9% saline, followed by either 10% formalin (for pSTAT3 detection) or 4% PFA (for pERK detection) in 0.1 M PBS as described above. This time point is optimal to determine activation of second messenger signaling molecules such as pSTAT3 and pERK in neonates (Walker et al., 2007). After blood collection, perfusion, and post-fixation, brains were transferred to 20% sucrose (in 0.1 M PB) overnight. The following day, brains were frozen on dry ice and stored at −80°C until processed for immunohistochemistry. Brains were collected as 50 μm floating sections in cryoprotectant (30% ethylene glycol, 20% glycerol, 37.5% diethylpyrocarbonate H_2_O, 8% NaCl, 0.02% KCl, 1.4% Na_2_HPO_4_, and 0.24% KH_2_PO_4_ in dH_2_O, pH 7.4) and stored at −20°C until staining. All primary and secondary antibodies, fixation conditions, and blocking solutions for each IHC are summarized in Table 2.

**TABLE 2:** List of antibodies and immunohistochemistry procedures.

**Primary AB**	**Fixative**	**Blocking solution**	**Primary AB dilution**	**Secondary AB**
Rabbit anti-pSTAT3 (Cell Signaling #9131S)	10% formalin	4% Normal goat serum 0.4% Triton-X100 1% BSA (in TBS-T)	1:500	Biotinylated goat-anti-rabbit 1:500 (Vector Labs Cat# BA-1000)
Goat anti-CART (R&D Systems #AF163)	10% formalin	4% Normal horse serum 0.4% Triton-X100 1% BSA (in TBS-T)	1:100	Donkey-anti-goat Alexa488 1:200 (Life Technologies #A11055)
Mouse anti-GAD (Millipore MAB#5406)	10% formalin	2% Normal goat serum 0.04% Triton-X100 1% BSA (in TBS-T)	1:500	Goat-anti-mouse Alexa568 1:500 (Invitrogen #A11031)
Rabbit anti-pERK (Cell Signaling #4370S)	4% PFA	3% Normal horse serum 0.3% Triton-X100 1% BSA (in TBS-T)	1:200	Donkey-anti-rabbit Cy3 (Jackson ImmunoResearch #711-165-152) 1:500
Goat anti-ORX-A (Santa Cruz SC#8070)	4% PFA	3% Normal horse serum 0.3% Triton-X100 1% BSA (in TBS-T)	1:200	Donkey anti-goat Alexa 488 (Life Technologies #A11055) 1:200

*AB, antibody; BSA, bovine serum albumin; ORX-A, orexin A; PFA, paraformaldehyde; TBS-T, 0.1 M Tris buffered saline containing 0.1% Tween20.*

### Single or Double Immunohistochemistry (IHC) of Neonatal LHA After Leptin Treatment

In the first experimental series, we examined leptin-induced activation of pSTAT3 in LHA neurons (single pSTAT3 IHC). Sections were brought to room temperature (RT), washed 3 × 5 min in 0.1 M Tris-buffered saline containing 0.1% Tween 20 (TBS-T) and incubated in 0.5% H_2_O_2_ and 0.5% NaOH in TBS-T for 20 min at RT. Sections were washed again (4 × 5 min in TBS-T) and placed in 0.3% glycine in TBS-T for 10 min at RT, followed by a wash (3 × 5 min in TBS-T) and another incubation with 0.03% SDS in TBS-T for 10 min at RT. After washing, sections were placed in a blocking solution (Table 2) for 1 h at RT, treated for 15 min with avidin-biotin (Vectastain, Burlingame, CA, United States) at RT, and washed before incubation with the primary rabbit anti-pSTAT3 antibody (Cell Signaling 9131S, 1:500 dilution in blocking solution; New England Biolabs, Ipswich, MA, United States) for 1 h at RT and then overnight at 4°C. The following day, sections were washed and placed in biotinylated goat-anti-rabbit secondary antibody (Vector Cat. No: BA-1000, 1:500 in blocking solution; Vector Labs, Burlingame, CA, United States) for 2 h at RT. Next, sections were washed and placed in streptavidin conjugated to Cy3 (Jackson ImunoResearch 016-160-084, 1:1000 in blocking solution; Jackson ImmunoResearch, West Grove, PA, United States) for 2 h at RT. Sections were washed (3 × 5 min in TBS-T), coverslipped using mounting medium with DAPI (Vector Labs, Burlingame, CA, United States), dried and stored at 4°C prior to imaging.

In the second experimental series, we performed double IHC to identify the phenotype of leptin-activated neurons in the LHA using pSTAT3 IHC with either CART or GAD67 as a marker of GABAergic neurons. The protocol remained the same through the avidin and biotin treatment and thereafter, tissue sections were placed in the appropriate blocking solution (Table 2) for 1 h at RT. Sections were washed and incubated in either mouse-anti-GAD67 (Millipore MAB5406, 1:500 in blocking solution, MilliporeSigma, Burlington, MA, United States) or goat-anti-CART (R&D Systems AF163, 1:100 in blocking solution; R&D Systems, Minneapolis, MN, United States) primary antibody for 2 h at RT, and overnight at 4°C. The following day, sections were washed and placed in the appropriate secondary antibody: goat-anti-mouse Alexa568 for GAD67 (A11031 Invitrogen, 1:500 in blocking solution; Life Technologies, Carlsbad, CA, United States) or donkey-anti-goat Alexa488 for CART (Life Technologies A11055, 1:200 in blocking solution; Thermo Fisher Scientific Corporation, Carlsbad, CA, United States). Sections were stained for pSTAT3 detection as described above, coverslipped, and stored at 4°C prior to analysis.

In the third experimental series, we performed double IHC for detection of pERK and ORX-A. Sections were brought to room temperature (RT), washed 3 × 5 min in 0.1 M TBS-T and treated with 1% NaOH + 1% H_2_O_2_ in TBS-T for 30 min at RT, followed by TBS-T washes (3 × 5 min). Sections were treated with 0.3% glycine in TBS-T for 10 min at RT, washed and treated with 0.03% SDS in dH_2_O, washed again (TBS-T, 3 × 5 min), and treated with 100% methanol at −20°C. Following washes, the sections were incubated in blocking solution (Table 2) for 1 h at RT. Thereafter, sections were incubated in rabbit anti-pERK primary antibody (Cell Signaling 4370S, 1:200 dilution in blocking solution; Cell Signaling Technology, Danvers, MA, United States) for 1 h at RT and overnight at 4°C. The following day, sections were washed and incubated for 2 h in Cy3 donkey-anti-rabbit secondary antibody (Jackson ImmunoResearch 711-165-152, 1:500 dilution in blocking solution; Jackson ImmunoResearch, West Grove, PA, United States) at RT. Sections were washed and treated with a goat anti-ORX-A primary antibody (Santa Cruz SC8070, 1:200 dilution in blocking solution; Santa Cruz Biotechnology, Dallas, TX, United States) overnight at 4°C. On the final day, sections were washed, treated with Alexa 488 donkey anti-goat secondary antibody (Life Technologies A11055, 1:200 dilution in blocking solution; Thermo Fisher Scientific Corporation, Carlsbad, CA, United States) for 2 h at RT, washed as previously described, and coverslipped using mounting medium with DAPI (Vector Labs, Burlingame, CA, United States), and stored in the dark at 4°C until imaged.

### Image Acquisition and Signal Quantification

For fluorosphere-injected brains with proper placement in the LHA ORX-A cell field, serial z-stack tiling images of the DMH and VMH were taken at 20x using an Olympus BX63 microscope with a step size of 0.35 mm to visualize retrogradely transported beads. Images were reconstructed using CellSens Software (version 1.12 to 1.15; Olympus Canada Inc., Toronto, ON, Canada) and the VMH and DMH regions were contoured using nuclear DAPI staining. Projections originating from the DMH and VMH were quantified using the optical fractionator probe for unbiased stereology (Microbright Field Bioscience, MBF, Williston, VT, United States). Within these regions, clusters of beads clearly showing a neuronal shape (either contained in the axon or clustered in the cell body) were counted as one projection. In the VMH region, a counting frame of 100 μm X 100 μm was chosen with a 200 μm X 200 μm sampling grid and a dissector height of 30 μm. Counted projections in the VMH began at −1.72 mm and extended to −3.36 mm from the bregma. For the DMH region, the entire region was counted with a 150 μm X 150 μm counting frame and a 20 μm dissector height. The DMH region began at −3.00 mm and extended to −3.48 mm from the bregma (Paxinos and Watson, 2005). The Gundersen coefficient of error (*m* = 1) (Gundersen et al., 1999) was 0.09 ± 0.008 and 0.14 ± 0.009 for the VMH and DMH, respectively. Projection density was calculated using the estimated population of cells (# of cells containing fluorospheres) and the volume of the counted regions (MBF). The average volume for the VMH was 5.82 ± 0.238 × 10^8^ μm^3^ (CD) and 6.26 ± 0.223 × 10^8^ μm^3^ (HFD). The average volume for the DMH was 0.503 ± 0.037 × 10^8^ μm^3^ (CD) and 0.535 ± 0.051 × 10^8^ μm^3^ (HFD). Additionally, 4X serial images were taken of the injection site in the LHA and used to normalize the density of projections counted to the maximum volume of beads injected. This was determined for each brain using the LHA section with highly fluorescent beads at the most ventral point, and was considered the reference point of the bead injection. In this particular section, we calculated the volume of bead injection by multiplying the surface area (obtained using ImageJ Software; ImageJ 1.50i; National Institutes of Health, United States) by the thickness of the section (measured with stereology, MBF). This value was used to normalize the density of projections per animal by dividing the density of projections counted from the DMH and VMH (cells/mm^3^) by the volume of beads (mm^3^) injected. To obtain a higher-resolution visualization of both immunostaining and DMH/VMH projections, selected sections were imaged using an Olympus FV1200 confocal microscope with a motorized stage (Olympus Canada Inc., Toronto, ON, Canada). Images were acquired using 2X line averaging, and were 2048 pixels X 2048 pixels. Both the 405 nm and 543 nm lasers were used, with emission barrier filters BA430-470 and BA550-660, respectively.

Images of immunostained tissue sections (for pSTAT3, pSTAT3/CART, pSTAT3/GAD67, or pERK/ORX-A) were taken with an Olympus BX63 microscope at either 20X or 40X using an Olympus DP80 camera and motorized stage (Olympus Canada Inc., Toronto, ON, Canada). For immunostained tissue, multichannel tiling images were taken at 20X. All tiled images were reconstructed using CellSens Software. For the detection and quantification of pERK and ORX-A double IHC, colocalized and single stained cells were counted using ImageJ. Both ORX-A and pERK cell density (number of cells/mm^2^ area) were calculated and the percent of ORX-A cells co-localizing with pERK was determined. Nuclear pSTAT3 signal in single IHC experiments was quantified using the QuPath software (Bankhead et al., 2017). We evaluated the precision and reliability of the QuPath quantification by performing parallel manual counting of all sections for pSTAT3 analysis on PND10 pups. The correlation between both modes of counting was significant (*p* < 0.001) with an *r*^2^ = 0.3899 (*n* = 141 determinations).

### Plasma Leptin Concentrations

Plasma leptin levels were measured using a specific rat leptin ELISA kit (Sigma Aldrich, St. Louis, MI, United States) with a detection range of 30–8,000 pg/ml and following the manufacturer’s indications. Plasma samples (50 μl) of neonates were assayed for all cohorts in duplicates and within the same assay plate if possible.

### Statistical Analysis

For projection analysis, a one-way ANOVA was used to compare the normalized density of projections between diet (CD vs. HFD) groups. For the quantification of immunofluorescent signal in the LHA after leptin treatment, we performed a two-way ANOVA using diet (CD vs. HFD) and treatment (saline vs. leptin injection) as between factors. Analysis of variance was followed by *post hoc* Tukey HSD tests as appropriate. Values are represented as mean ± SEM and statistical differences with *p* < 0.05 are considered significant.

## Results

### Maternal High Fat Diet Effects on Caloric Intake, Pup Body Weight and Leptin Concentrations

**FIGURE 2:**
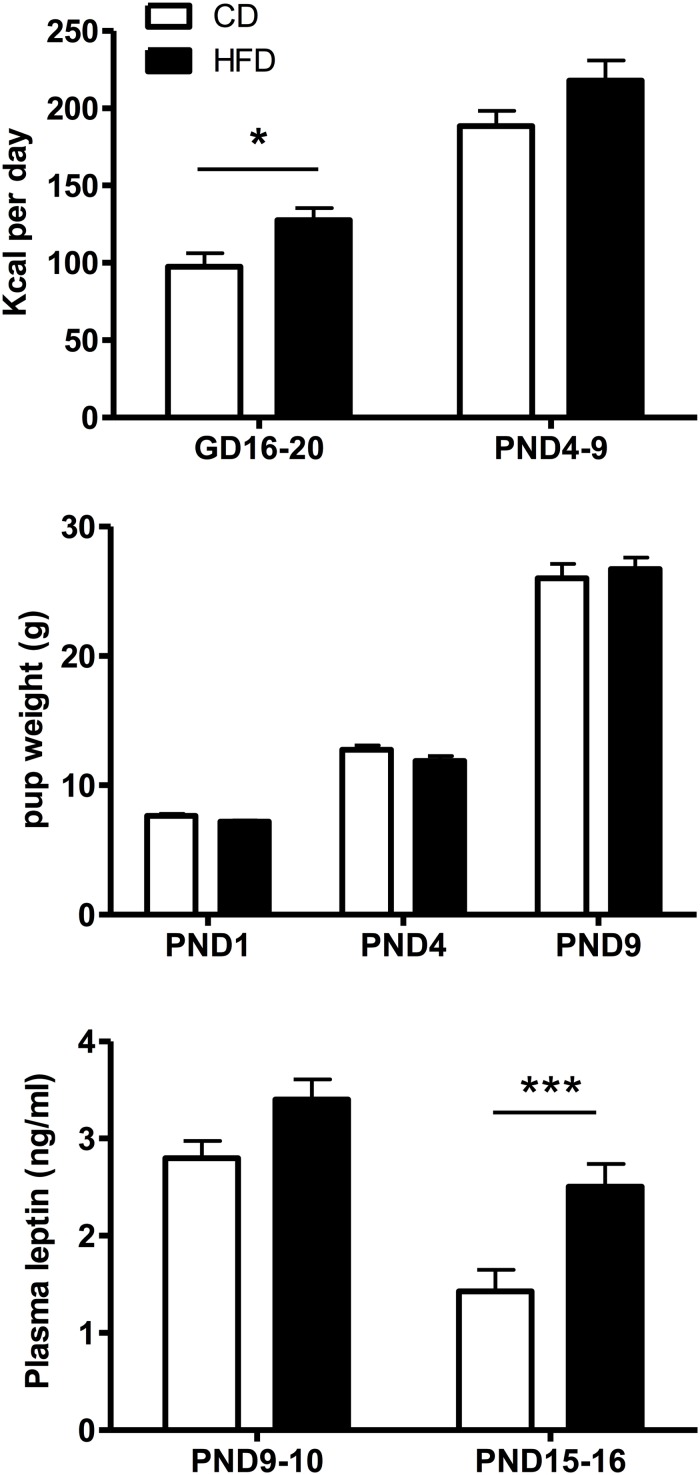
Top: Time-related changes in maternal caloric intake (Kcal/day) pre- and postpartum in mothers fed a control (CD, *n* = 11) or high fat (HFD, *n* = 10) diet. Caloric intake was calculated over an interval of 4–5 days during gestation (GD16-20) or postpartum (PND4-9) for four experimental series. Middle: Age-related changes in CD or HFD pup weight between PND1 and 9. Bottom: Changes in plasma leptin levels as a function of age and diet exposure in PND9-10 (*n* = 23–24) or PND15-16 (*n* = 20) pups. Values represent the mean ±SEM. *p < 0.05; ***p < 0.001 compared to the CD group (*post hoc* Tukey HSD test).

Maternal Kcal intake (Figure 2, top panel) varied significantly as a function of diet (*F*(1,19) = 7.84, *p* = 0.011) and time (*F*(1,19) = 125.3, *p* < 0.001) and *post hoc* Tukey HSD test showed that Kcal intake was significantly (*p* = 0.021) increased in HFD compared to CD mothers during gestation, but not when measured postpartum (*p* = 0.061). Pup weight during the first 10 days of life did not change significantly with the maternal diet (Figure 2, middle panel) in line with the lack of significant diet effect on leptin levels on PND9-10 pups (Figure 2, bottom panel). In contrast, plasma leptin levels on PND15-16 were significantly increased in HFD compared to CD-fed pups (*p* = 0.002, *t*-test).

### Density of Hypothalamic (DMH and VMH) Projections to the LHA ORX-A Neuronal Field

**FIGURE 3:**
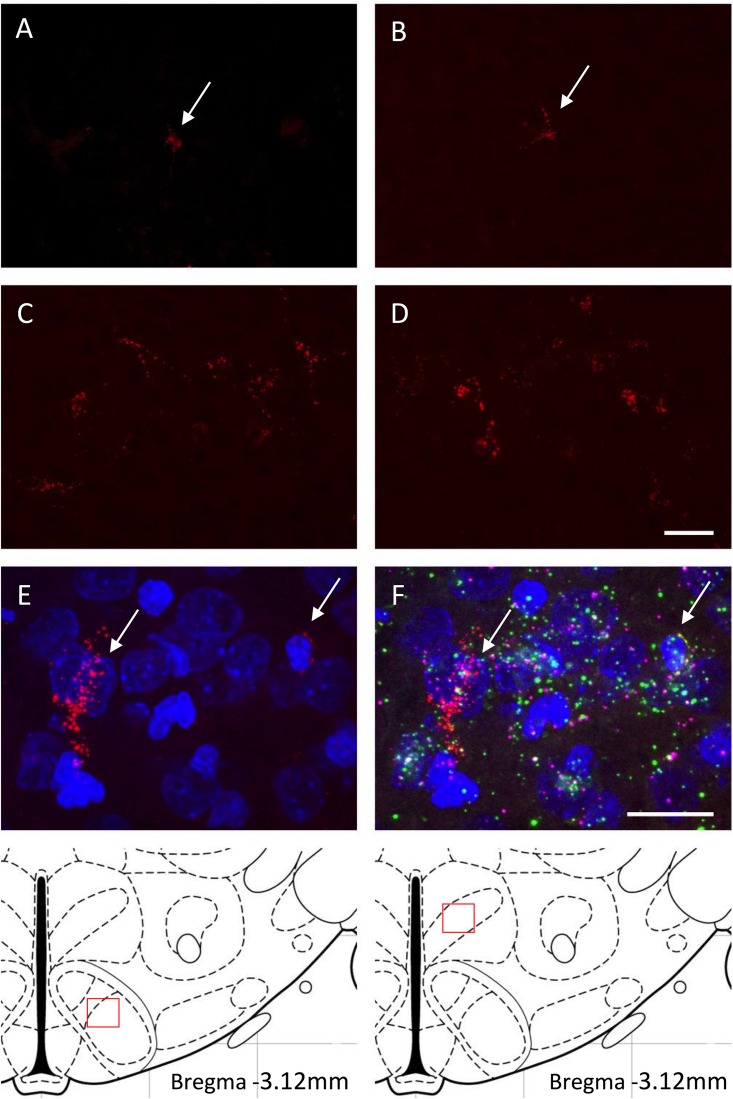
**(A–D)** Retrogradely labeled neurons of the VMH **(A,C)** and DMH **(B,D)** with fluorescent microspheres in PND9-10 rat pups from either the control diet **(A,B)** or the high fat diet **(C,D)** groups. Fluorescent microspheres were injected on PND5-6 in the ORX-A neuronal cell field of the lateral hypothalamic area (LHA). White arrows on the top photographs indicate few projection neurons that contain microspheres in the CD pups. Higher density of labeled cells is observed in the HFD pups. A schematic of the area analyzed is provided on the bottom of the figure. All projection photographs **(A–D)** were taken at 40X magnification and the scale bar represents 20 μm. Phenotypic identification of VMH neurons projecting to the ORX-A region of the LHA in PND9-10 rat pups by RNAscope **(E,F)**. Subpopulations of VMH neurons displaying retrograde microspheres **(E)** were identified by expression of either BDNF (green) or SF-1 (pink) mRNA molecules **(F)**. Photographs for the RNAscope analysis were taken on an Olympus FluoView FV1200 confocal microscope at 60X magnification. Scale bar for **(E,F)** panels represents 20 μm.

**FIGURE 4:**
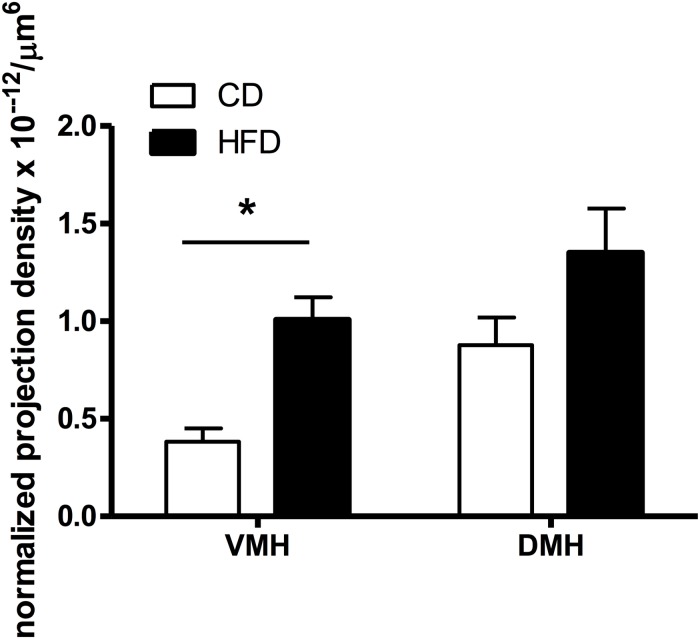
Quantification of the density of VMH and DMH neurons expressing retrogradely transported microspheres from the ORX-A field of the LHA in CD or HFD PND9-10 pups. Projection density was calculated using the estimated population of cells (# of cells containing fluorospheres) and the volume of the counted regions (#cells/μm^3^), further normalized by the volume of beads injected (μm^3^) in the LHA region for each injected animal. The resulting normalized density of projections (cells/μm^6^) is compared between CD (*n* = 8) and HFD (*n* = 10) pups (see “Materials and Methods” for detailed description). Values represent the Mean ±SEM. *p < 0.05 compared to CD group (one way ANOVA).

We determined whether maternal HFD modified the density of hypothalamic projections to the ORX-A field of the LHA in the offspring using injections with fluorescent retrograde microbeads to the LHA ORX-A cell field at PND5-6 (Figure 1). Retrograde labeling in the DMH and VMH was measured 4 days later (PND9-10). These nuclei were chosen because of their important role in metabolic regulation and because the maturation of their projections to the LHA is relatively early compared to afferents from the ARC for instance. By PND9-10, projections to the LHA were observed from both the DMH and VMH in animals of both diet groups, thus confirming that axons from these regions have reached the LHA ORX-A cells at least by this age. As illustrated in Figure 3, projection density originating from either the VMH (Figures 3A,C) or the DMH (Figures 3B,D) was higher in pups from HFD mothers. When the normalized projection density was quantified (Figure 4), we found a significant effect of diet on the projection density originating from and observed in the VMH (*F*(1,10) = 4.79, *p* = 0.05), with HFD pups exhibiting a higher density compared to CD pups. The projection density originating from the DMH also tended to be increased with exposure to the HFD, although the values did not reach statistical significance (*F*(1,10) = 3.24, *p* = 0.10). Other regions such as the mPFC also displayed modest labeling, that was not quantified. Labeling of the ARC nucleus was very sparse with occasional cells displaying beads, consistent with the later onset of ARC to LHA projections around PND10-12 (Bouret et al., 2004b).

We next wanted to determine whether the origin of early projections to the LHA ORX-A field in the VMH was cell population specific and we performed VMH RNAscope analysis for SF-1 and BDNF neuronal populations on sections exhibiting retrograde fluorospheres. As illustrated in Figure 3F, projections to the LHA in PND9-10 pups originated from either neurons expressing BDNF alone or from those co-localizing SF-1 with BDNF.

### Leptin-Induced pSTAT3 Activation in PND10 and PND16 Neonates

**FIGURE 5:**
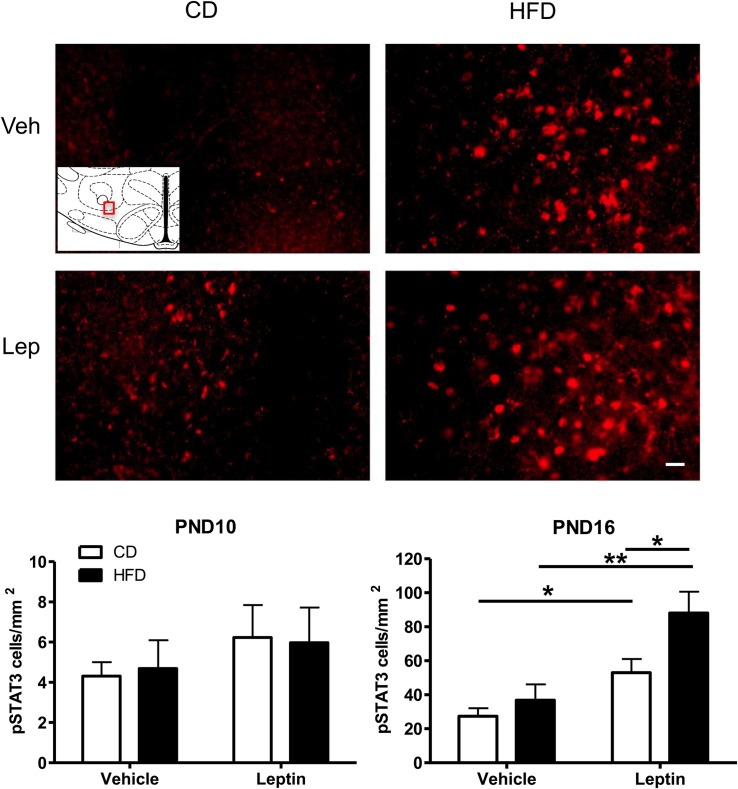
Changes in Phospho-STAT3 (pSTAT3) immunoreactivity in the ORX-A field of the LHA in PND16 rat pups receiving either vehicle or leptin (3 mg/kg bw, ip) injection 60 min earlier. Expression of pSTAT3 staining after either treatment is compared between CD (left) or HFD (right) pups. The insert indicates the LHA region that is used for illustration (Bregma level = –3.12 mm). Photographs were taken at a 20X magnification and the scale bar represents 20 μm. Bottom panels: Quantification of immunoreactive pSTAT3 cell density (#cells/mm^2^) in the LHA of PND10 (*n* = 5/treatment group) and PND16 (*n* = 4–5/treatment group) pups from either CD or HFD litters. Values are Mean ±SEM. *p < 0.05; **p < 0.01 *post hoc* Tukey HSD test.

Given the increase in projections to LH ORX-A cells in HFD pups, we next examined the ability of peripheral leptin injection (3 mg/kg BW i.p.) to modify responsiveness to leptin in the LHA-ORX-A field on PND10 and later, at the onset of independent feeding on PND16. Figure 5 illustrates the changes in pSTAT3 immunostaining in the LHA of PND16 pups following either vehicle or leptin administration in CD and HFD pups. On PND10, there was no significant treatment or diet effect and no treatment X diet interaction on the expression of pSTAT3 immunostaining in the LHA ORX-A field as shown in an earlier study (Gjerde et al., 2016). In contrast, on PND16, HFD pups exhibited significantly higher pSTAT3 activation in response to leptin compared to CD pups. ANOVA for pSTAT3 positive cell density showed a significant effect of diet (*F*(1,14) = 6.53, *p* = 0.023) and treatment (*F*(1,14) = 19.60, *p* < 0.001), but no significant diet X treatment interaction. Tukey HSD *post hoc* tests showed that the effect of diet was significant for the leptin-treated group (*p* = 0.013), but not the vehicle-treated group. Increase in pSTAT3 immunostaining after leptin injection was significant both for the CD (*p* = 0.044) and the HFD (*p* = 0.0015) groups, showing that the onset of functional LHA cellular responses to leptin on PND16 can be modulated by maternal diet exposure.

**FIGURE 6:**
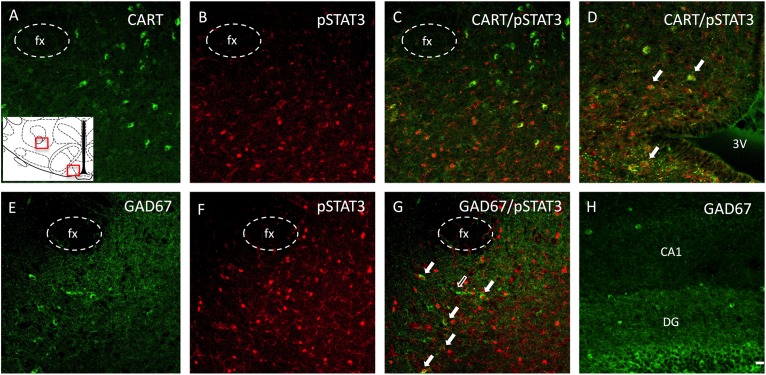
Phenotypic identification of neurons expressing pSTAT3 in the ORX-A field of the LHA in a leptin-treated PND16 rat pup by double immunohistochemistry. **(A)** CART immunoreactive neurons are found lateral to the fornix (fx), **(B)** pSTAT3 positive neurons, **(C)** lack of colocalization between CART and pSTAT3 in the LHA. **(D)** In contrast to the LHA, pSTAT3 highly colocalizes with CART neurons in the arcuate nucleus. Another set of sections were examined for GABA neurons (GAD67), **(E)**, pSTAT3 neurons **(F)**, and neurons colocalizing both GAD67 and pSTAT3 **(G)** in the LHA. Few cells in the neonatal LHA express GAD67 **(E)**, but some of these co-localize with pSTAT3 expression [**(G)**, full arrows]. **(H)** The dentate gyrus (DG) of the hippocampus expresses a higher density of GAD67 neurons compared to the LHA **(E)** at this age. All photographs were taken at bregma level = –3.12 mm, at 20X magnification and the scale bar represents 20 μm. The insert represents the areas that are depicted in this figure for the LHA and the arcuate nucleus. 3V, third ventricle; fx, fornix; DG, dentate gyrus; CA1, pyramidal CA1 region of the hippocampus.

In order to phenotype leptin activated neurons in the LHA of PND16 pups, we performed double immunocytochemistry for pSTAT3 and CART or GAD67 (GABAergic neurons). As indicated in Figures 6A–C, there was no pSTAT3 co-localization with CART in either CD or HFD groups, as these were distinct cell populations. However, as a control, we found quite extensive co-localization between pSTAT3 and CART in the arcuate region of the hypothalamus on PND16 (Figure 6D). In contrast to CART, we observed a small percentage of pSTAT3 neurons that co-localized with GAD67 on PND16 (Figures 6E–G), but only in leptin treated pups (Percentage of colocalization: CD-Lept = 1.59 ± 1.17, *n* = 4; HFD-Lept: 1.81 ± 0.65, *n* = 5). Immunostaining for GAD67 was generally lower in the LHA compared to the hippocampus (Figure 6H) on PND16 and the number of GAD67-positive cells was not modified by either diet or leptin treatment (number of GAD cells LHA: CD-Veh: 67.3 ± 9.47, *n* = 4; CD-Lept: 69.3 ± 11.3, *n* = 4; HFD-Veh: 60.8 ± 12.9, *n* = 5; HFD-Lept: 77.8 ± 13.9, *n* = 5).

### Indirect Activation of LHA ORX-A Neurons in the Neonatal LHA

**FIGURE 7:**
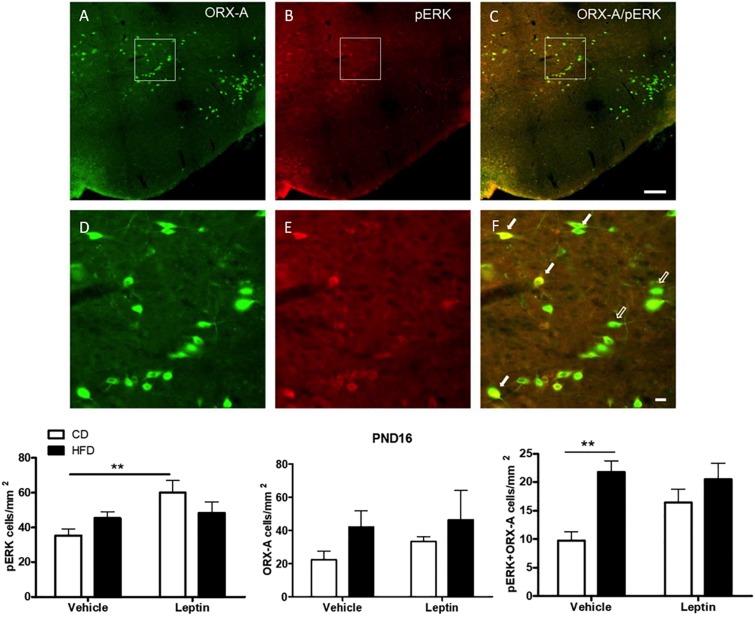
Immunohistochemical detection of ORX-A **(A)**, pERK **(B)** cells in the LHA of a leptin-treated PND16 pup. Phospho-ERK expression is observed in some ORX-A neurons [**(C)**, full arrows], but not in others [**(C)**, empty arrows] at this age. Details of individual and co-localized immunoreactive neurons are provided in panels **(D–F)**. Photographs were taken at a 20X magnification and the scale bar represents 200 μm **(A–C)** or 20 μm **(D–F)**. Quantification of immunostained neurons in the LHA. **p < 0.01 Posthoc Tukey HSD test.

Although LHA ORX-A neurons do not harbor leptin receptors, they might still be activated indirectly by intra-hypothalamic or extra-hypothalamic leptin-sensitive projections, some of these being potentially increased by the maternal HFD. Thus, we examined leptin-induced indirect ORX-A activation by evaluating the co-localization of ORX-A with pERK (a marker of neuronal activation) in pups (Figure 7). At PND10, there was no significant effect of diet or treatment on the number of pERK-positive and ORX-A cells, or the proportion of ORX-A cells co-localizing with pERK (data not shown). On PND16, there was a significant effect of treatment (*F*(1,18) = 6.54, *p* = 0.0198) on pERK density and a close to significant diet X treatment interaction (*p* = 0.058). Leptin treatment increased pERK-positive cells in the LHA of CD (*p* = 0.0062), but not HFD pups. Neither diet nor treatment modified the number or ORX-A cells at this age. However, early diet had a significant effect to increase pERK and ORX-A co-localization (ANOVA: diet effect *F*(1,18) = 12.22, *p* = 0.0025), in particular in vehicle-treated pups (*p* = 0.0016). Leptin’s effect to increase pERK/ORX-A colocalization was close to significant in CD pups (*p* = 0.065), but was not modified in the HFD pups.

## Discussion

The aims of the present study were to determine whether perinatal maternal feeding with a HFD could modify the establishment of hypothalamic projections to the LHA in neonatal pups and alter the sensitivity of LHA neurons to the metabolic hormone leptin, which is increased by maternal high fat feeding and exerts neurodevelopmental actions in neonatal rodents (Bouret et al., 2004a). Our results demonstrate that maternal high fat feeding increases the number of VMH projections to the LHA in pups already by PND10 and increases direct responsiveness of LHA neurons to exogenous leptin administration at the time of onset of independent feeding around PND15-16. Interestingly, leptin-activated neurons that exhibit pSTAT3 immunoreactivity in neonates are not co-expressing CART and might constitute only a small subset of GABAergic LHA neurons. These results suggest that maternal diet has a significant effect on the hypothalamic control and responsiveness of the LHA during a developmental time period that is critical for the establishment of feeding circuits.

The LHA is a large heterogeneous structure and a critical hub between homeostatic and hedonic feeding centers. We focused more specifically on afferents to the ORX-A field of the LHA because this neuronal population directly regulates dopamine (DA) neurons in the VTA (Moorman and Aston-Jones, 2010; Leinninger et al., 2011; Muschamp et al., 2014; Godfrey and Borgland, 2019). Although these cells do not express the LepRb, they are regulated by leptin-sensitive afferent regions (Louis et al., 2010; Goforth et al., 2014) and accordingly modulate DA reward. To evaluate the density of projections to this area, we stereotaxically injected fluorescent retrograde tracer beads to the ORX-A field in CD and HFD PND5-6 pups and examined hypothalamic VMH and DMH labeling on PND9-10. We chose this experimental time course because this window during hypothalamic maturation potentially captured the development of afferents and provided enough time for substantial labeling in afferent nuclei. Though labeling was observed in various regions, we focused on afferents from the DMH and VMH because these nuclei express leptin receptors early in life (Bouret, 2010) and regulate food intake in the adult (Choi et al., 2013; Stuber, 2016; Jeong et al., 2017). Furthermore, ARC connections reach the DMH by PND6 (Bouret et al., 2004a) and the VMH at an unknown stage during development. While the ARC is highly sensitive to leptin by the first week of postnatal development (Bouret, 2010; Naef et al., 2014), the direct ARC-LHA fibers do not mature until PND12 (Bouret et al., 2004a). Thus, the DMH and VMH are likely to relay leptin-sensitive input from the ARC to the LHA neurons before PND12, although it is currently unclear when projections from the DMH and VMH reach the LHA. Here, we examined if these projections are sensitive to maternal diet and mature earlier than the ARC-LHA connections.

Both HFD and CD pups showed retrograde labeling in the DMH and VMH by PND10, which confirmed that at least some of the projections between these regions and LHA ORX-A neurons are already present at this age. When compared to CD pups, HFD pups displayed significantly more retrograde labeling in the VMH and tended to have more labeling in the DMH. Our results suggest that a maternal HFD either accelerates the onset of projection development or increases the number of fibers projecting to the LHA. In contrast to VMH and DMH, we observed little to no labeling in the ARC at PND10, confirming the absence of direct ARC-LHA projections at this age. In the adult, the VMH preferentially innervates the medial portion of LH ORX-A cells, while the DMH innervates both medial and lateral areas of the ORX-A neuronal field (Yoshida et al., 2006). Our data does not indicate any difference in projection density in these regions between medially- vs. laterally-injected pups, however we have a small sample size for each group which prevents a full examination of labeling as a function of LHA injection site. By combining retrograde labeling and RNAscope techniques, we showed that VMH neurons projecting to LHA in neonates did not constitute a unique cell population, but that both Steroidogenic factor 1 (SF-1) and brain-derived neurotrophic factor (BDNF) expressing VMH neurons projected to the LHA. Signature SF-1-expressing VMH neurons harbor leptin receptors and are known to increase thermogenesis via activation of sympathetic output rather than regulating food intake (Dhillon et al., 2006; Kim et al., 2011). Also present in the VMH are unidentified neuronal populations that regulate food intake rather than energy expenditure, which may be sensitive to leptin and project to the LHA (Choi et al., 2013). Early increases in circulating leptin concentrations in pups from the HFD mothers might be important to activate these neurons and promote the development and maintenance of their projections to other hypothalamic areas. Although not significant, maternal HFD also tended to increase projection density from the DMH in PND10 pups.

Interestingly, we observed an increased fiber density in the VMH in PND10 HFD pups despite the fact that at this age, circulating leptin levels were not significantly increased compared to CD pups. While this does not eliminate an important effect of leptin on the development of projections, it suggests that other factors present in or triggered by the HFD exposure, such as BDNF (Genzer et al., 2016) might promote neurite outgrowth in neonatal life. It is also possible that specific fatty acids (FA) present in the maternal HFD and transmitted to the offspring through the milk may be responsible for increased neuronal proliferation and/or the increased density of projections that we observed in HFD neonates. In adult mice, supplementation with dietary unsaturated omega-3 fatty acids was shown to induce hypothalamic cell proliferation specifically in the ARC nucleus (Nascimento et al., 2016) and other *in vitro* studies found that docosahexaenoic acid (DHA) induces neurite outgrowth and promotes neuronal differentiation in isolated hippocampal neurons (Calderon and Kim, 2004). Given that our diet was relatively rich is unsaturated fatty acids (63%) this might explain why, by PND10, HFD pups displayed increased hypothalamic projections despite no differences in weight gain or leptin concentrations compared to CD pups. Alternatively, changes in dietary fat could also modify the number of hypothalamic VMH or DMH neurons by altering their survival. Although hypothalamic neurogenesis (mostly ARC) ends by the second week of gestation in rodents, proliferation extends until the late fetal and perinatal periods, precisely when mothers were exposed to the HFD (Bouret, 2017). One limitation to our study is that in our experiments, retrograde fluorescent microbeads were injected into the region of the LHA containing ORX-A cells, but they may have been taken up by synapses formed with other cell types as well. For instance, we have previously shown that ORX-A neurons are intermingled with Neurotensin neurons in neonates (Gjerde et al., 2016) and thus, microbeads may have been transported by afferent fibers innervating these or other cells in this area.

Given the diet-induced increase in afferents from the VMH to the ORX-A cell field of the neonatal LHA, we next examined whether leptin-induced neuronal activation of LHA neurons was enhanced by the maternal diet. In contrast to ORX-A neurons, many cell types in the LHA harbor LepRb, including CART, GABAergic cells co-localizing with Neurotensin for instance (Berthoud and Münzberg, 2011; Leinninger, 2011; Goforth et al., 2014) and other phenotypically uncharacterized cells. As observed earlier (Gjerde et al., 2016), leptin-induced pSTAT3 expression was substantially higher on PND15-16 compared to PND10, when neither diet nor treatment effect was significant. On PND15-16, leptin treatment induced a robust pSTAT3 response in both CD and HFD pups and a larger response in HFD pups, suggesting that the LHA has become more responsive to leptin signaling as a result of either higher circulating levels of leptin and/or increased density of projections to the LHA. This result might be unique to the developmental period and/or represents early re-programming of hypothalamic pathways since Fos responses to central leptin administration in the LHA of adult male rats was reduced by adult exposure to a HFD (Alsuhaymi et al., 2017). Robust leptin-induced pSTAT3 immunostaining on PND15-16 was also observed in the ARC, VMH, and DMH, possibly reflecting heightened leptin receptor expression in these areas in the HFD pups and/or increased sensitivity of intracellular pathways at this age. Leptin-induced pSTAT3-positive neurons in the LHA co-localized modestly with GAD67, a marker of GABA neurons, but not with CART. This was surprising as adult CART neurons of the LHA express LepRb mRNA (Berthoud and Münzberg, 2011; Farzi et al., 2018) and we observed robust CART immunostaining and co-localization with pSTAT3 in the ARC in young neonates. However, this could also represent an early adaptation to favor an anabolic state during development because, in contrast to LHA CART, stimulation of ARC CART reduces energy expenditure in adult mice (Farzi et al., 2018). The lack of leptin-induced activation of CART in the neonatal LHA indicates that the orexigenic and motivational actions of CART (Somalwar et al., 2017) in this structure might not be fully developed by the second week of life.

In older pups (PND15-16), leptin-induced pSTAT3 activation was observed in a subset of LHA GABAergic neurons despite the fact that expression of GAD67 immunoreactivity was sparse in the LHA of either CD or HFD pups when compared to other regions such as the hippocampus. It is possible that leptin-responsive GABAergic cells in the LHA mature later than GABAergic cells in other regions and that GABAergic tone in the LHA is low during neonatal life. The mature LHA contains many subsets of GABAergic cells and some co-express LepRb and various neuropeptides (Leinninger et al., 2009; Berthoud and Münzberg, 2011; Leinninger, 2011). In adult mice, GABAergic LHA fibers innervate both VTA GABA and Dopamine neurons although preferential innervation of VTA GABA neurons results in disinhibition of DA neurons (Stuber and Wise, 2016). The low percentage of leptin-induced pSTAT3/GAD67 cells in both CD and HFD groups compared to the robust pSTAT3 activation observed in the same groups (Figure 5) suggests that other non-GABAergic cell populations are activated by leptin in neonates. Further experiments should better characterize these phenotypically unknown activated neurons and determine if they project to the VTA to regulate DA function.

Orexin-A cells of the LHA directly regulate VTA DA neurons, but they do not express LepRb (Goforth et al., 2014). These ORX-A cells are already functionally connected to the VTA by the end of the first week of age (Gjerde et al., 2016) and thus might be important to regulate the development of DA function (Naef et al., 2014). To examine the potential effect of maternal HFD on differential ORX-A activation, we used pERK as a marker of general neuronal activation in response to leptin-sensitive afferent projections to these neurons. Production of pERK is stimulated by leptin (Frühbeck, 2006; Kwon et al., 2016) and other ligands and is especially important during neonatal development due to its role in cell proliferation and survival (Zhang and Liu, 2002). On PND10 there was no effect of diet or leptin challenge on the number of pERK cells or the proportion of ORX-A cells co-localizing with pERK in the LHA (not shown). On PND15-16, Significant pERK responsiveness to leptin was observed in CD pups, but not in HFD pups. This was unexpected given the increased pSTAT3 responsiveness observed in this region after leptin administration in HFD pups (Figure 5). This results suggests that in HFD pups, pERK might not be an accurate indicator of leptin responsiveness. The maternal HFD dramatically increased pERK/ORX-A co-localization in vehicle-treated pups, suggesting a larger activation of ORX-A neurons after the stress of vehicle injection. However, it is unclear why we did not observe a significant increase in pERK/ORX-A colocalization after leptin injection, in particular in the HFD pups. It is possible that pERK activation reached a ceiling effect in HFD pups because of the high levels observed even after vehicle injection. Interestingly, the number of ORX-A neurons was not significantly altered by the maternal diet and this is consistent with other reports indicating that a maternal HFD during pregnancy (Poon et al., 2012) or neonatal leptin administration (Proulx et al., 2002) alters the expression of other appetite-regulated peptides but not ORX-A.

In summary, our experiments demonstrate that a maternal HFD provided during the last week of gestation and during lactation increases the density of VMH afferents to the LHA in neonatal PND10 offspring, and more specifically in the region where ORX-A cells are located. This is associated with increased responsiveness of LHA neurons to elevated leptin levels and could lead to potential dysregulation of VTA DA neurons since these are primarily regulated by LHA afferents. What remains to be determined is whether other metabolic signals also regulate LHA function during this critical developmental period and participate in the programming of the feeding circuitry. Because our study does not allow distinguishing between the prenatal and/or postnatal effects of the maternal dietary environment on the offspring, future experiments should establish more precise critical windows for maturation of these circuits. Further research in this area will greatly contribute to our understanding of the developmental origins of dysregulated feeding behavior and metabolic disease in adulthood.

## Data Availability Statement

The datasets generated for this study are available on request to the corresponding author.

## Ethics Statement

The animal study was reviewed and approved by the Douglas Institute Animal Care Committee, McGill University and followed ethical guidelines from the Canadian Council on Animal Care (CCAC).

## Author Contributions

LK and C-DW designed the study, analyzed the data, and wrote the manuscript. LK performed the experiments and collected all data together with HL and SV. ML helped in the analysis of the ORX-A/pERK data.

## Conflict of Interest

The authors declare that the research was conducted in the absence of any commercial or financial relationships that could be construed as a potential conflict of interest.
